# A Diagnostic Dilemma of a Case of Granulomatosis With Polyangiitis (GPA) Presenting With Thrombotic Vasculopathy

**DOI:** 10.7759/cureus.34479

**Published:** 2023-01-31

**Authors:** Aunchalee Jaroenlapnopparat, Peymaan Banankhah, Joseph Khoory, Chinmay Jani, Shiv Sehra

**Affiliations:** 1 Internal Medicine, Mount Auburn Hospital, Harvard Medical School, Cambridge, USA; 2 Rheumatology, Mount Auburn Hospital, Harvard Medical School, Cambridge, USA

**Keywords:** anca vasculitis, thrombotic vasculopathy, hemoptysis, wegener’s granulomatosis, gpa, granulomatosis with polyangiitis

## Abstract

Granulomatosis with polyangiitis (GPA) is a rare disease with a prevalence of about three in 100,000 persons in the United States. GPA is an antineutrophil cytoplasmic antibody (ANCA)-associated vasculitis affecting predominantly small-sized vessels. It can present with localized or systemic symptoms with multiple organ involvement, thus making diagnosis challenging. Common skin lesions in GPA are palpable purpura, petechiae, ulcers, and livedo reticularis. These lesions usually have underlying vasculitis with or without granuloma on histology findings. To date, there have been no previous reports about thrombotic vasculopathy in GPA before.

We present a case of a 25-year-old female who presented with intermittent joint pain for weeks, purpuric rash, and mild hemoptysis for a few days. A review of systems was notable for a 15-pound weight loss in one year. Physical examination was significant for a purpuric rash on the left elbow and toe, and left knee swelling and erythema. Presenting laboratory results were notable for anemia, indirect hyperbilirubinemia, mildly elevated D-dimers, and microscopic hematuria. Chest radiograph revealed confluent airspace disease. Extensive infectious workup was negative. A skin biopsy of her left toe revealed dermal intravascular thrombi without evidence of vasculitis. The thrombotic vasculopathy did not favor vasculitis but raised concern for a hypercoagulable state. However, extensive hematologic workup was negative. Bronchoscopy findings were consistent with diffuse alveolar hemorrhage. Later, cytoplasmic ANCA (c-ANCA) and anti-proteinase 3 (PR3) antibody titers were positive. Her diagnosis was unclear since both skin biopsy and bronchoscopy were nonspecific and inconsistent with her positive antibody results. The patient eventually underwent a kidney biopsy, which showed pauci-immune necrotizing and crescentic glomerulonephritis. Finally, a diagnosis of granulomatosis with polyangiitis was made based on the kidney biopsy and positive c-ANCA. The patient was treated with steroids and IV rituximab and discharged home with outpatient rheumatology follow-up.

Due to multiple signs and symptoms including thrombotic vasculopathy, there was a diagnostic dilemma requiring a multidisciplinary approach. This case highlights the importance of pattern recognition for the diagnostic framework of rare disease entities and the multidisciplinary collaborative efforts required to reach the final diagnosis.

## Introduction

Granulomatosis with polyangiitis (GPA) is a rare disease with a prevalence of about three in 100,000 persons in the United States [1]. GPA is one of antineutrophil cytoplasmic antibody (ANCA)-associated vasculitis, in which the body generates antibodies that attack small- to medium-sized vessels in the body. This process causes cascading inflammatory events in the affected tissues of the body, leading to multi-organ presentations such as hematuria, hemoptysis, and sinusitis.

The pathogenesis of the disease is not well understood but is considered to be multifactorial. Multiple immune abnormalities result in the overproduction of autoimmune antibodies, cytoplasmic ANCA (c-ANCA), which act against a protein called proteinase 3 (PR3) and induce neutrophil-mediated vascular injury. Vasculitis with or without granuloma formation on histology from skin lesions is the underlying manifestation of GPA. Necrosis of blood vessel walls is the result of inflammatory cytokines and other mediators induced by ANCAs, and T-cell hyperactivity, particularly CD4+ T-cells, is related to granuloma formation [2].

On the other hand, thrombotic vasculopathy has never been reported in GPA before. We present the first case of a patient with thrombotic vasculopathy who was ultimately diagnosed with GPA. Due to multi-organ symptoms including thrombotic vasculopathy, there was a diagnostic dilemma requiring a multidisciplinary approach. This case highlights the importance of pattern recognition for the diagnostic framework of rare disease entities and the multidisciplinary collaborative efforts required to reach the final diagnosis.

## Case presentation

**Figure 1:**
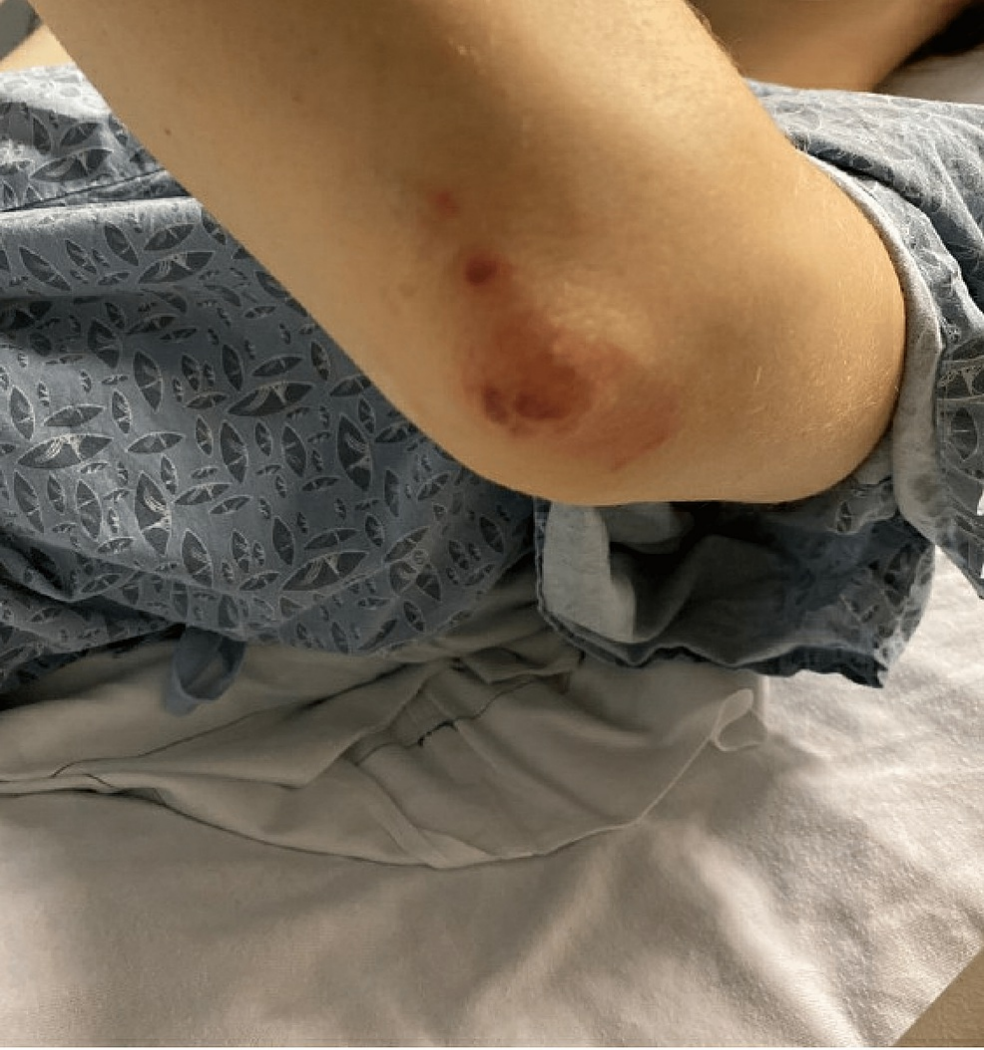
Purpuric rash on the left elbow

**Figure 2:**
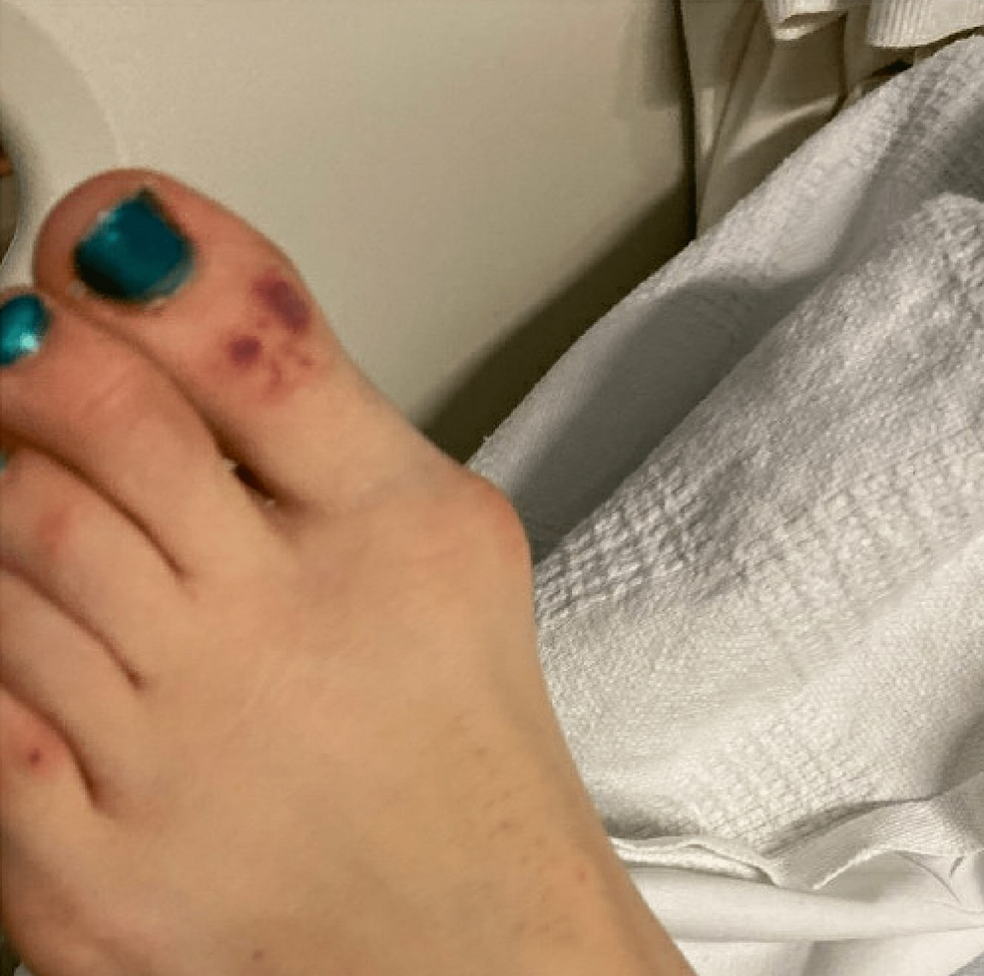
Purpuric rash on the left toe

A 25-year-old Caucasian female presented to the emergency department with intermittent joint pains for four weeks and hemoptysis. She reported first symptoms of difficulty making a fist, first with the right hand and then progressing to the left hand. She reported migratory joint pain that affected her bilateral elbows and shoulders. Each joint pain episode lasted approximately 36 hours before migrating to different joints. Three days before admission while she was traveling in the Midwest United States, she had three bouts of small-volume hemoptysis, approximately one teaspoon each time, which prompted her to come to the hospital. A review of systems was notable for an unexplained 15-pound weight loss in one year. She has a past medical history of migraine and a history of Achilles tendon infection in 2019. She took sarecycline daily for acne for two months before admission. She works with chimpanzees in the laboratory. Her physical examination was significant for non-blanchable purpuric rash on the left elbow and toes and swelling and erythema of the left knee (Figure 1 and Figure 2). Her nasal and otoscopic examination was unremarkable.

**Figure 3:**
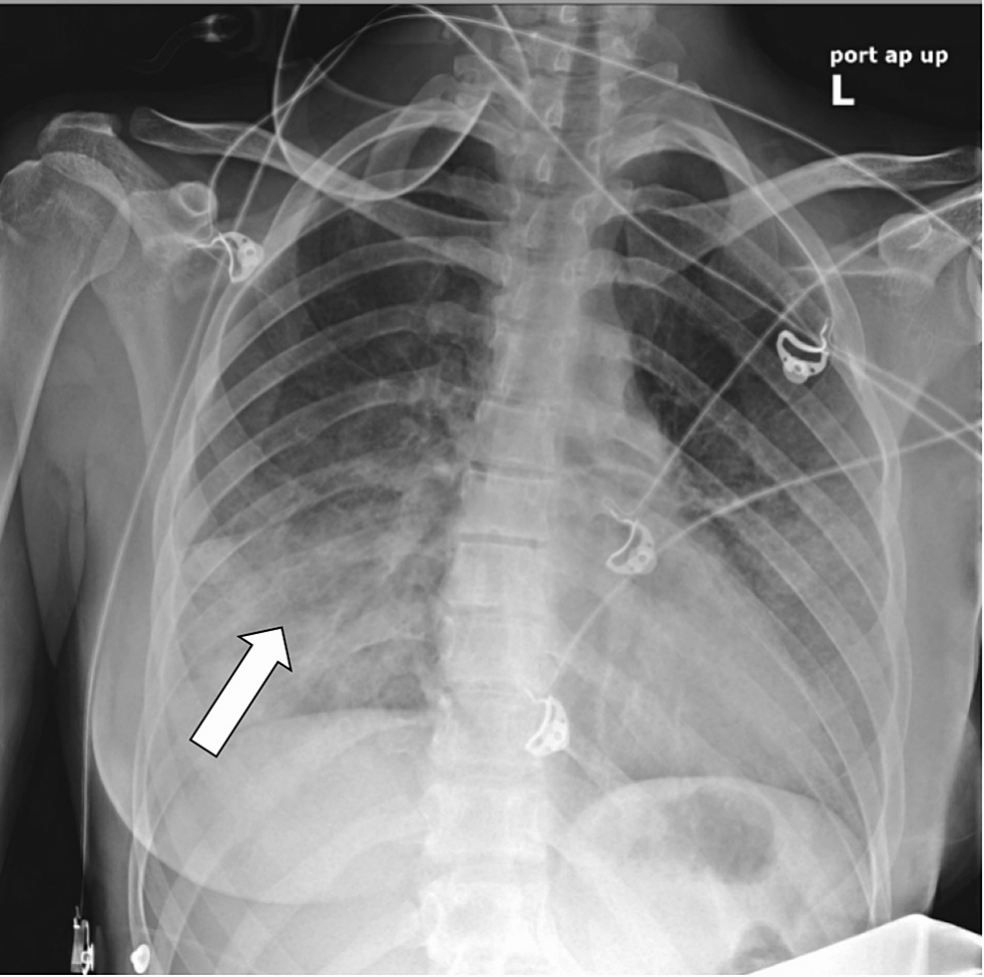
Chest X-ray showing confluent airspace disease, most prominent at the right lower lobe (arrow)

Her presenting blood investigation was notable for normocytic anemia, low iron levels, and hypereosinophilia (eosinophils 9%) (normal range: 0%-5%). She had mildly elevated total bilirubin and C-reactive protein (CRP). Her urine analysis was positive for ketones and red blood cells (RBCs). Initial chest X-ray showed confluent airspace disease, prominent in the lower lobe, and right greater than left (Figure 3).

In summary, the patient presented with migratory joint pain, purpuric rash, weight loss, anemia, low iron levels, indirect hyperbilirubinemia, hypereosinophilia, microscopic hematuria, and mild hemoptysis. Due to the subacute course and systemic involvement, an autoimmune process such as systemic vasculitis was highly suspected. Within the differential, infectious and paraneoplastic processes were also considered. Infectious workup, including human immunodeficiency virus (HIV), hepatitis B and C panels, Histoplasma, parvovirus, *Mycoplasma pneumoniae*, tuberculosis, *Pneumocystis jirovecii*, *Cytomegalovirus*, herpes simplex virus (HSV)-1, and HSV-2, were all negative. Transthoracic echocardiogram did not show evidence of endocarditis or atrial myxoma. Vasculitis diseases such as ANCA-associated vasculitis and immune complex vasculitis were highly suspected. We sent autoimmune laboratory results for ANCA and other autoimmune diseases that could be related to immune complex vasculitis such as cryoglobulins vasculitis, lupus, and rheumatoid arthritis. Goodpasture’s syndrome and sarcoidosis were also in the differential diagnosis, which could be obtained by bronchoscopy. Anti-glomerular basement membrane (GBM) antibody was sent for possible Goodpasture’s syndrome. The sarecycline that the patient used before admission has never been reported to be associated with ANCA-associated vasculitis before.

Finally, a skin biopsy was suggested and did not show vasculitis. However, it revealed dermal blood vessels with intravascular thrombi without evidence of vasculitis. Because of the increased risk of hypercoagulable states from intravascular thrombi and concern for hemolytic anemia, hematology was consulted. The patient underwent multiple hematology workups, including prothrombin time (PT), activated partial thromboplastin time (aPTT), fibrinogen, beta-2 glycoprotein, cardiolipin antibody, cryoglobulins, lupus anticoagulant, protein C, protein S, antithrombin III, and factor V Leiden, which were all negative. She had mildly elevated D-dimer (592 ng/mL) (normal range: <281 ng/mL), which did not support any specific diagnosis. Her direct and indirect Coombs tests were negative, suggesting that this was less likely to be autoimmune hemolytic anemia. Her peripheral blood smear did not show evidence of schistocytes, and her lactic dehydrogenase (LDH), haptoglobin, was negative, which helped rule out microangiopathic hemolytic anemia.

**Figure 4:**
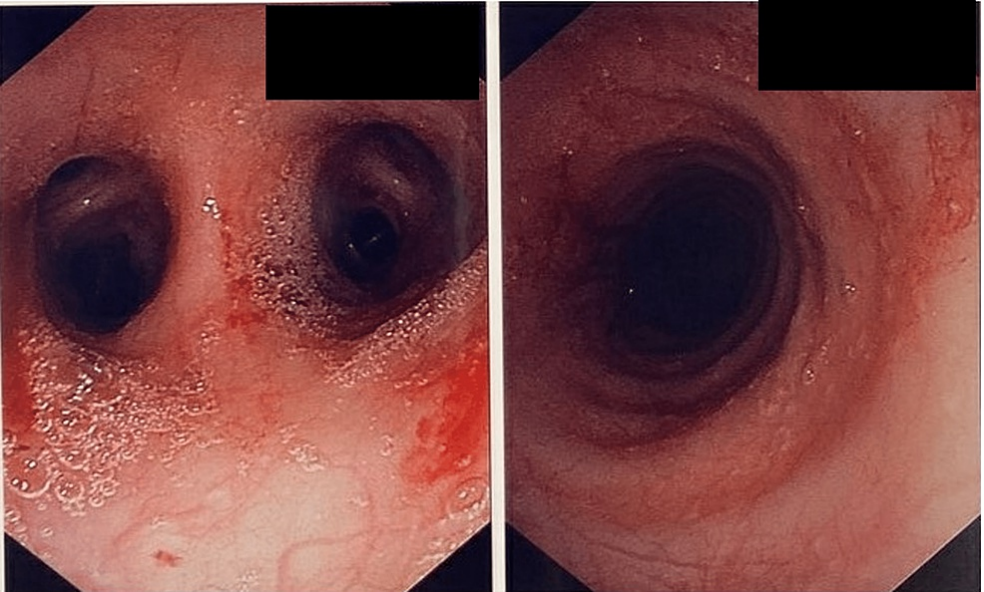
Bronchoscopy revealing erythematous lesions on the tracheal mucosa

Pulmonology was consulted due to an unclear diagnosis, and bronchoscopy with bronchoalveolar lavage (BAL) and transbronchial biopsy was performed. The bronchoscopy revealed erythematous lesions on the tracheal mucosa (Figure 4). Cytology from BAL was negative for malignancy. Biopsy showed airway wall fibrosis, chronic inflammation, and scattered eosinophils with no definitive evidence of vasculitis. Fragments of lung parenchyma with hemosiderin-laden macrophages and focal intra-alveolar fibrin were seen. The findings were consistent with diffuse alveolar hemorrhage. Hypersensitivity pneumonitis was less likely due to a negative test. Acute eosinophilic pneumonia was less likely due to low numbers of eosinophils from BAL.

Later in her hospitalization, the autoimmune laboratory results came back. Antinuclear antibody (ANA), anti-GBM antibody, and cyclic citrullinated peptide (CCP) were negative. Rheumatoid factor was 19.5 IU/mL (normal range: <12 IU/mL). Her anti-PR3 antibody and cytoplasmic ANCA (c-ANCA) titer were positive at 1:320. Given the positive anti-PR3 antibody and c-ANCA, ANCA-associated vasculitis, especially GPA, microscopic polyangiitis (MPA), was the most likely diagnosis. Eosinophilic granulomatosis with polyangiitis (Churg-Strauss syndrome) is more associated with anti-myeloperoxidase (MPO) antibody than PR-3 antibody and thus less likely to be the cause. The patient was treated with 1 g methylprednisolone for three days before transitioning to prednisone 60 mg daily for presumptive vasculitis.

Nephrology was consulted for microscopic hematuria. Her urine sediment showed multiple white blood cells, dysmorphic RBC, and RBC casts raising concerns of renal involvement from systemic disease. Since the diagnosis remained unclear even after a skin biopsy and a bronchoscopy, a kidney biopsy was performed. Finally, a kidney biopsy showed pauci-immune necrotizing and crescentic glomerulonephritis involving about 20% of intact glomeruli and minimal (1% of glomeruli) chronic changes of the parenchyma (focal global glomerulosclerosis). A diagnosis of granulomatosis with polyangiitis with articular, pulmonary, renal, and skin involvement was made. Due to her fertile age, rituximab was chosen instead of cyclophosphamide to avoid side effects as it was not an inferior option [3]. The patient eventually received one dose of IV rituximab at 375 mg/m^2^ and was discharged with outpatient rheumatology follow-up.

## Discussion

ANCA-associated vasculitis is slightly more common in men than in women and is usually found in middle-aged adults. Our patient has had an earlier onset since she was a 25-year-old woman. More than 90% of cases occur in the Caucasian population, just like our patient, although this might be an overestimation based on health inequalities such as the availability of serology tests and healthcare resources [4]. Early diagnosis and intervention lead to the clinical remission of 75% of patients and is a vital strategy to reduce morbidity and mortality rates [5]. Several studies identified environmental risk factors and mostly pointed to dust and farm exposure [6]. In our case, the patient has extraordinary animal exposure as she works with monkeys in the laboratory. In a previous study, GPA significantly associates with horse exposure, but there was no evidence of GPA risk associated with monkey exposure [7].

Clinical manifestations of GPA at onset include a nonspecific prodrome of symptoms such as fever, fatigue, weight loss, myalgia, and arthralgia, which can last for days to months before organ-specific symptoms presented in our case. The respiratory tract and kidneys are classically involved, just like in our case, with hemoptysis and microscopic hematuria. The underlying histopathology in GPA is necrotizing non-caseating granulomatous vasculitis, which helps differentiate it from other medium-sized vasculitis. In 2022, the European League Against Rheumatism (EULAR)/American College of Rheumatology (ACR) released the classification criteria for GPA. The criteria with weights will be calculated in total scores, and a diagnosis of GPA can be made if the cumulative score was ≥5 points. The criteria include various presentations such as nasal involvement, c-ANCA or anti-PR3 antibody positivity, and findings on chest imaging and biopsy [8]. In our patient, she had a total score of 1 before a kidney biopsy (c-ANCA or anti-PR3 antibody positivity (+5) and high eosinophil count (-4)); thus, she did not meet GPA criteria. Given the unclear diagnosis at that time, a kidney biopsy was done and showed pauci-immune glomerulonephritis on biopsy. Our patient received the diagnosis after fulfilling the histological definition of GPA, which included visualization of necrotizing granulomatous inflammation within the upper or lower respiratory tract, vasculitis within small- to medium-sized blood vessels, glomerulonephritis, and positive ANCA antibodies [9]. Of note, after starting steroids for presumptive vasculitis, her eosinophil level had decreased to normal, which supported the clinical picture of GPA.

Common dermatologic manifestations in GPA are purpura, urticaria, and livedo reticularis. The underlying histopathological findings that are commonly found in ANCA-associated vasculitis are leukocytoclastic vasculitis or perivascular granulomatosis with or without granuloma [10]. Our patient initially presented with a purpuric rash, but a skin biopsy interestingly showed thrombotic vasculopathy. Thrombotic vasculopathy was found in diseases such as warfarin- and heparin-induced skin necrosis, which are unlikely in her case due to no exposure. Given normal hematologic laboratory work, protein C and protein S deficiency syndrome, disseminated intravascular coagulation syndrome, cryoglobulinemia, homocysteinemia, macroglobulinemia, and antiphospholipid syndrome were less likely. We believe that this is the first case report of GPA that manifested with thrombotic vasculopathy.

The respiratory tract is involved in 95% of GPA cases [11]. Pulmonary nodules, patchy infiltration, diffuse infiltration, and pleural effusion can be seen from lung imaging, and patients can present with various upper to lower respiratory tract symptoms such as tracheitis, hemoptysis, pleuritis, and hilar lymphadenopathy. GPA is unique to other ANCA-associated vasculitis diseases as it has the propensity to affect the upper respiratory tract, resulting in sensorineural hearing loss, otitis, sinusitis, nasal crusting, and nasal ulceration [12]. Our patient presented with mild hemoptysis that could be caused by ANCA-associated vasculitis or immune complex vasculitis, which requires a biopsy result to show a difference. Goodpasture syndrome can manifest with hemoptysis and positive ANCA; thus, biopsy is helpful while awaiting anti-GBM antibody results. Progressive renal disease has been identified as a leading cause of death in GPA [13]. Approximately 80% of GPA cases had kidney involvement, which can take up to two years to develop. Patients usually present with hematuria, non-nephrotic range proteinuria, or rapidly progressive glomerulonephritis (RPGN). Our patient had RBC cast and dysmorphic RBC without alterations in renal function. Other organ system involvement includes neurological (mononeuritis multiplex, paresthesias, and ophthalmoplegia) and ophthalmic (conjunctivitis, corneal abrasion, corneal ulcer, and retro-orbital pseudotumor), which did not present in our patient [14]. GPA usually tests positive for c-ANCA (anti-PR3 antibody). However, 10% of GPA cases can have negative ANCA. Moreover, false-positive results can occur, particularly in systemic infections such as subacute infective endocarditis [15]. The specificity of immunoassays also depends on other concurrent symptoms. For example, patients with sinusitis and pulmonary hemorrhage have 90% specificity to have GPA if they test positive for c-ANCA. On the other hand, patients with limited organ involvement can have negative ANCA as high as 40% [16]. Hence, the definitive diagnosis is more accurately obtained by tissue biopsy.

The treatment goal for GPA is to suppress immune activity. For the induction phase in the active and severe cases as in our case, steroids are used with immunosuppressants such as rituximab or cyclophosphamide. For the maintenance phase, agents such as rituximab, methotrexate, azathioprine, leflunomide, or mycophenolate mofetil can be used [17]. Plasmapheresis can be considered in patients with rapidly progressive renal disease and can be used as an adjunct to steroids and cyclophosphamide to reduce hemodialysis rates. Conversely, plasmapheresis does not seem to reduce overall mortality rates [18]. In our case, the patient received pulse steroids and rituximab for the induction phase to suppress active disease in the setting of diffuse alveolar hemorrhage. Rituximab was chosen over cyclophosphamide due to a more favorable side effect profile. The patient was subsequently transitioned to the maintenance phase with prednisone. She did not undergo plasmapheresis because of no evidence of progressive renal failure.

## Conclusions

In summary, we present a young female presenting with multisystem symptoms of unclear cause, who was ultimately diagnosed with GPA on renal biopsy. This case was unique in that, in addition to pulmonary and renal involvement, her skin biopsy showed thrombotic vasculopathy, a finding that had not been previously reported in GPA, making the diagnostic evaluation particularly challenging. Although it is considered a rheumatologic disease, making a diagnosis of vasculitis often requires a multidisciplinary approach to rule out other contributory causes. Eventually, all her laboratory work was negative, except for c-ANCA and anti-PR3 antibody, indicating that all her presentations were secondary to ANCA vasculitis. The patient improved after receiving pulse steroids and rituximab. This case highlights the importance of pattern recognition for the diagnostic framework of rare disease entities and the multidisciplinary collaborative efforts required to reach this diagnosis.
